# Genome-wide identification and expression analysis of nucleotide-binding site leucine-rich repeat genes associated with downy mildew resistance in spinach

**DOI:** 10.7717/peerj.21599

**Published:** 2026-08-14

**Authors:** Jiao Yang, Man Yuan, Xuemei Wang, Xiaoli Wang, Chenxi Xu, Xiaofeng Cai

**Affiliations:** Shanghai Collaborative Innovation Center of Plant Germplasm Resources, College of Life Sciences, Shanghai Normal University, Shanghai, China

**Keywords:** Spinach, NBS-LRR, Downy mildew, Gene family, Phylogenetic analysis

## Abstract

**Background:**

Downy mildew is a destructive disease that causes severe losses in spinach (*Spinacia oleracea* L.) yield and quality. Nucleotide-binding site-leucine-rich repeat (NBS-LRR) genes are core regulators of plant innate immunity and play critical roles in defense against pathogen infection. However, the characteristics of NBS-LRR family genes in spinach and their specific functions in downy mildew resistance remain unclear, limiting the development of resistant spinach cultivars.

**Methods:**

Using available spinach genome data, we identified NBS-LRR family members *via* bioinformatics approaches. We classified these members into subclasses, analyzed their chromosomal distribution, conserved motifs, and within-clade functional similarity. Combining genome-wide association study (GWAS) results, we selected candidate genes associated with downy mildew resistance. We detected the expression patterns of these candidate genes in different tissues and between resistant and susceptible spinach genotypes. We also analyzed the expression dynamics of candidate genes in resistant spinach (Whale) under downy mildew stress and performed protein–protein interaction (PPI) analysis to identify core proteins involved in the resistance response and their potential disease-related associations.

**Results:**

We identified 114 NBS-LRR family members in spinach, which were categorized into coiled-coil (CC) domain—NBS (40 members), CC-NBS-LRR (27 members), NBS (19 members), NBS-LRR (12 members), and other subclasses. These genes were asymmetrically distributed across chromosomes, with enrichment on chromosomes 1 and 3 (accounting for 26.3% and 28.1% of the total, respectively). NBS-LRR members shared conserved motifs, with motifs 2 and 12 being the most highly conserved; genes within the same phylogenetic clade showed functional similarity. Ten candidate genes were selected through integration with GWAS results. These candidates exhibited tissue-specific expression (*e.g.*, *SoarcTNL1* was highly expressed in petioles and roots) and differential expression between resistant and susceptible materials (*SoarcRN1* showed the highest expression in resistant US78). Under downy mildew stress, candidate genes were more strongly upregulated in resistant Whale, with expression inflection points or peaks at 6 and 8 days post-inoculation (dpi) with expression inflection points or peaks at 6 and 8 dpi. PPI analysis identified core proteins *SoarcRN1* and *SoarcNL8* in high-confidence networks. This study clarifies the characteristics of spinach NBS-LRR genes and their associations with downy mildew resistance, laying a critical foundation for subsequent gene function research and resistant spinach breeding.

## Introduction

Plants in natural environments are constantly challenged by diverse pathogens, and disease develops when plants lack effective resistance to these invaders (Fonseca & Mysore, 2019; Zvereva & Pooggin, 2012). Plant defense against pathogens relies on innate immunity, which comprises three main layers: basal defense, resistance (*R*) gene-mediated defense, and RNA silencing (RNA interference) (Jones & Dangl, 2006). Cell surface pattern recognition receptors (PRRs) recognize conserved pathogen-associated molecular patterns (PAMPs) and activate pattern-triggered immunity (PTI) to limit pathogen infection (Thomma, Nürnberger & Joosten, 2011). PRRs are plasma membrane-localized and typically include receptor-like kinases (RLKs) or receptor-like proteins (RLPs) lacking kinase domains. In response, pathogens secrete effector molecules to evade or suppress PTI, leading to effector-triggered susceptibility (ETS). In turn, plants have evolved intracellular nucleotide-binding leucine-rich repeat receptors (NLRs), usually encoded by *R* genes, to detect these effectors and activate effector-triggered immunity (ETI) (Lacaze & Joly, 2020). Pathogens may further evolve to diversify or lose effectors to escape ETI. Meanwhile, various signaling pathways (similar to those involved in resistance to other biotrophic pathogens) are activated, resulting in the production of specific defense molecules (Jones & Dangl, 2006; Moffett, 2017). Although PTI and ETI rely on distinct receptor recognition mechanisms, they share common defense signaling events, such as reactive oxygen species (ROS) accumulation, synthesis of antimicrobial compounds and defense hormones, transcriptional reprogramming, and activation of defense-related genes. Abnormal accumulation or overactivation of immune impairs plant growth and development; thus, plants tightly regulated immune receptor activity (Teixeira et al., 2019; Garcia-Ruiz, 2019).

NBS-LRR genes are defined by three characteristic domains: a variable N-terminal domain, a central nucleotide-binding site (NBS) domain, and a C-terminal leucine-rich repeat (LRR) domain (Takken & Tameling, 2009). Based on the presence or absence of an N-terminal Toll/Interleukin-1 receptor (TIR) domain, NBS-LRR genes are further divided into TIR-NBS-LRR (TNL) and non-TIR-NBS-LRR (non-TNL) subclasses (Takken & Tameling, 2009). In the plant innate immune system, receptors encoded by NBS-LRR genes localize in the cytoplasm and specifically recognize pathogen-secreted effectors (*via* direct or indirect recognition). This recognition triggers complex downstream signaling cascades, often resulting in rapid localized cell death (hypersensitive response, HR) at infection sites and other biochemical changes that limit pathogen proliferation (Chisholm et al., 2006). The central NBS domain (also called as the NB-ARC domain) contains highly conserved motifs required for ATP/GTP binding and hydrolysis. The NBS domain is followed by tandem LRR domains, which provide recognition specificity for pathogen effectors (Innes, 2004). Other domains, such as zinc finger or RPW8 domains, may replace the coiled-coil (CC) domain at the N-terminus, and genes carrying these domains are generally classified as CC-NBS-LRR (CNL) genes (Sukarta, Slootweg & Goverse, 2016). Recent studies have demonstrated the roles of TIR and CC domains in pathogen recognition and downstream signaling (Williams et al., 2014). The distribution of TNL and CNL genes is species-specific: dicotyledonous plants harbor both subclasses, while monocotyledonous plants lack TNL genes (Shao et al., 2016; Sagi, Deokar & Tar’an, 2017).

Spinach (*Spinacia oleracea* L., 2n = 2×= 12) is a nutrient-dense leafy vegetable rich in vitamins and minerals. It is cultivated in more than 60 countries worldwide for its edible dark green leaves (Kandel et al., 2019). China, the United States, Iran, Japan, Turkey, and Indonesia are major spinach-producing nations, with China being the largest producer and consumer (https://www.fao.org/faostat/en/#data, 2025). In recent years, rising living standards have increased spinach consumption and raised demands for its nutritional quality. Like many other crops, spinach production is threatened by biotic stresses (diseases, pests, weeds) and abiotic stresses (salinity, drought, high temperature). Among these, downy mildew (caused by *Peronospora effusa*) is the most destructive disease, severely reducing global spinach yield and quality (Correll et al., 2011; Ribera et al., 2020). The control of fungal pathogens mainly relies on synthetic fungicides such as carbendazim, imidacloprid, and thiabendazole (Köhl, Kolnaar & Ravensberg, 2019). However, most conventional fungicides pose potential health risks to humans, and an increasing number of fungicide-resistant strains have been reported over recent decades (Calvo et al., 2019; Testempasis et al., 2020). Introgressing NBS-LRR resistance genes from wild relatives is a major strategy for breeding downy mildew-resistant spinach varieties, while using loss-of-function alleles of susceptibility genes offers a durable resistance breeding approach (Chisholm et al., 2006).

Although genome-wide identification of NBS-LRR genes has been reported in several vegetable crops, systematic characterization of the NBS-LRR family in spinach—including subclass classification, chromosomal distribution, conserved motif architecture, and phylogenetic relationships-remains incomplete. A recent study by She et al. (2026) conducted a pan-NLRome analysis of spinach and identified candidate downy mildew resistance genes, focusing on the discovery of novel resistance loci and their application in breeding. However, this study did not systematically characterize the NBS-LRR family in terms of subclass classification, chromosomal distribution, conserved motif architecture, and phylogenetic relationships. Furthermore, no studies (including She et al., 2026) have integrated genome-wide association studies (GWAS) loci for downy mildew resistance with NBS-LRR family analysis to validate candidate resistance genes, nor have they profiled the spatiotemporal expression patterns and pathogen-induced dynamics of NBS-LRR genes in resistant and susceptible spinach genotypes. The lack of such data hinders the understanding of molecular mechanisms underlying spinach downy mildew resistance and slows the molecular breeding of resistant cultivars.

To fill these critical knowledge gaps, this study used bioinformatics to identify and characterize NBS-LRR family members from the spinach reference genome (Cai et al., 2021). We combined GWAS results to screen candidate NBS-LRR genes associated with downy mildew resistance, analyzed their tissue-specific expression, expression divergence between resistant and susceptible materials, and expression dynamics under *P. effusa* stress, and predicted PPI networks to identify core resistance-related proteins. This work clarifies the structural and functional characteristics of spinach NBS-LRR genes and their regulatory roles in downy mildew resistance, providing a solid basis for gene functional validation and molecular breeding of downy mildew-resistant spinach. Importantly, this study complements the work of She et al. (2026) by providing a comprehensive framework for understanding NBS-LRR gene function, which will facilitate the validation and application of resistance genes in spinach breeding.

## Materials & Methods

### Identification of NBS-LRR family members in spinach

To systematically and comprehensively identify members of the spinach NBS-LRR gene family, the Hidden Markov Model (HMM) profile of the conserved NBS domain (PF00931) was downloaded from the Pfam database (http://pfam.xfam.org/). This HMM profile was used to search for candidate NBS-LRR proteins against the spinach genome, protein sequences, and annotation files using HMMER 3.0 software with an *E*-value cutoff of 1e−5. Redundant sequences were removed using CD-HIT software (sequence identity threshold = 90%). The remaining candidate sequences were submitted to the NCBI Conserved Domain Database (NCBI-CDD) (https://www.ncbi.nlm.nih.gov/Structure/cdd/wrpsb.cgi) for conserved domain analysis. Sequences lacking complete NBS domains or other key domains were discarded by integrating results from Pfam and NCBI-CDD. In addition, other domains (CC, LRR, RPW8, TIR) in the candidate sequences were confirmed based on CDD annotations.

### Analysis of gene structure, protein physicochemical properties, and functional domains

The exon-intron structures of NBS-LRR genes were extracted from the spinach genome annotation file (GFF format) and visualized using TBtools software (Chen et al., 2020). The physicochemical properties of the encoded amino acid sequences (molecular weight, isoelectric point, amino acid composition) were analyzed using the ExPASy ProtParam tool (https://web.expasy.org/protparam/). Subcellular localization of the proteins was predicted using the Euk-multi-2 tool (http://www.csbio.sjtu.edu.cn/bioinf/euk-multi-2/). Conserved proteins domains of the proteins were confirmed using the NCBI-CDD database. Conserved motifs were identified using the MEME suite (https://meme-suite.org/meme/) with the following parameters: number of motifs = 15, motif width = 6–50 amino acids. Promoter sequences (2,000 bp upstream of the start codon) were extracted using TBtools and uploaded to the PlantCARE database (http://bioinformatics.psb.ugent.be/webtools/plantcare/html/) to predict cis-acting elements. The results were visualized using TBtools combined with genome annotation files.

### Phylogenetic analysis of NBS-LRR family

Full-length sequences of 114 NBS-LRR protein were aligned using ClustalW with default parameters, and a phylogenetic tree was constructed in MEGA-X using the neighbor-joining method (NJ), and the bootstrap value was set to 1,000 to fully and comprehensively calculate the phylogenetic relationship of the NBS-LRR family. The NJ trees were visualized using Interactive Tree of Life (iTOL) (https://itol.embl.de/). Evolutionary clades with bootstrap values below 70% were noted in tree analysis. To explore potential interacting proteins, protein–protein interaction networks were predicted using STRING v11 software (Szklarczyk et al., 2019) with two confidence score thresholds: 0.15 (low confidence, for exploratory analysis) and 0.9 (high confidence, for robust biological inferences). A low confidence threshold (0.15) was used to initially explore potential interaction networks and identify a broad range of candidate interacting proteins, which provides a foundation for future functional validation. However, strong biological conclusions regarding protein interactions are based primarily on the high-confidence PPI network (confidence threshold = 0.9), as this threshold minimizes false-positive interactions and ensures greater reliability. PPI results were visualized using Cytoscape 3.8.2 software (Shannon et al., 2003).

### Plant materials and sampling

Spinach germplasms preserved in the Plant Germplasm Resource Center of Shanghai Normal University were used in this study, and all the experiments were conducted using the facilities at this center. Seeds were sown in plug trays filled with a 1:1 (v/v) mixture of peat soil and perlite and germinated in an artificial climate chamber. When seedlings developed two true leaves, uniform seedlings were transplanted into pots and grown in the same climate chamber with the following conditions: photoperiod of 10 h light/14 h dark, temperature of 21 °C (light)/18 °C (dark), light intensity of ∼5,000 lx, and relative humidity of ∼70%.

Samples were collected at three stages: (1) When the first true leaf reached 4–6 cm in length, leaves were sampled from different downy mildew-resistant and susceptible genotypes. (2) At the flowering stage of germplasm sp73 (susceptible material), roots, stems, functional leaves, functional leaf petioles, bolting leaves, and bolting petioles were collected. (3) At 60 days after sowing, leaf samples were collected from spinach germplasms with diverse agronomic traits (*e.g.*, leaf color, leaf shape, bolting time) were collected from the off-campus greenhouse of the Plant Germplasm Resource Center. All samples were immediately frozen in liquid nitrogen and stored at −80 °C until use.

### Preparation and inoculation of downy mildew pathogen

The downy mildew pathogen (*Peronospora effusa*) was purified through successive single-lesion isolation. The pathogen inoculation protocol used in this study is a modification of the method described by She et al. (2018). Specifically, She et al. (2018) used a spray inoculation method with a spore concentration of 1 × 10^4^ spores mL^−^^1^ and applied a 24-hour dark treatment post-inoculation. In our study, we used a spot-inoculation method with a higher spore concentration (1 × 10^5^ spores mL^−^^1^) to ensure consistent infection, and omitted the dark treatment to improve the specificity of plant–pathogen interactions (avoiding dark-induced stress on spinach seedlings). All other aspects of the protocol (pathogen purification, suspension preparation, and post-inoculation culture conditions) were consistent with She et al. (2018). The pathogen suspension was filtered through a seven-layer sterile filter paper to remove debris and concentrated by centrifugation (8,000 rpm, 10 min) to obtain a high-concentration spore suspension (1 × 10^5^ spores mL^−^^1^). The suspension was inoculated onto the first true leaf (4–6 cm in length) of spinach seedlings using a one mL pipette tip, with two inoculation points per leaf. After inoculation, seedlings were placed in a greenhouse at 20 °C and 100% relative humidity without dark treatment (to avoid the adverse effects of darkness on spinach growth and improve the specificity of the plant–pathogen interaction). Subsequently, the seedlings were cultured at 15–20 °C. From 0 to 10 days post-inoculation (dpi), leaf discs (one cm in diameter) centered on the inoculation points were collected, rinsed with sterile water, and pooled (10 discs per sample). All samples were immediately frozen in liquid nitrogen and stored at −80 °C.

### RNA isolation, cDNA synthesis, and qRT-PCR analysis

Total RNA was extracted from all samples using TRIzol reagent (Invitrogen, USA) according to the manufacturer’s instructions. RNA integrity was checked by 1% agarose gel electrophoresis, and RNA concentration and quality were determined using a NanoDrop 2000 spectrophotometer (Thermo Fisher Scientific, Waltham, MA, USA). Genomic DNA contamination was removed using DNase I (TaKaRa, Kyoto, Japan). First-strand cDNA was synthesized using the TaKaRa PrimeScript™ RT reagent Kit with gDNA Eraser (Perfect Real Time) according to the manufacturer’s protocol.

qRT-PCR was performed on an Applied Biosystems 7500 Real-Time PCR System (Thermo Fisher Scientific) with three biological replicates and three technical replicates per sample. Primers were designed using Primer-BLAST (https://www.ncbi.nlm.nih.gov/tools/primer-blast/) (Table S1), and the *So18s* gene was used as the internal reference. Use the specific primers designed by Shanghai Sangon Company, the PCR reaction system (20 μL) contained 10 μL of Bestar^®^ SybrGreen qPCR Mastermix (DBI Bioscience), 0.5 μL of forward primer (10 μM), 0.5 μL of reverse primer (10 μM), one μL of cDNA template (100 ng μL^−^^1^), and eight μL of ddH_2_O. The PCR program was as follows: 95 °C for 10 min, followed by 40 cycles of 95 °C for 15 s and 60 °C for 1 min. Melting curves showed single peaks with consistent Tm values, no primer dimers, or overlapping peaks, indicating reliable amplification. The relative expression levels of target genes were calculated using the 2^−ΔΔCt^ method.

Statistical analysis of qRT-PCR data was performed using one-way analysis of variance (ANOVA) followed by Tukey’s honestly significant difference (HSD) test for multiple comparisons, with a significance threshold of *P* < 0.05. Biological replicates (*n* = 3) were treated as independent samples, and technical replicates (*n* = 3) were averaged for each biological replicate to reduce experimental variation. *Z*-score normalization was used to standardize gene expression levels across different samples, which facilitates the comparison of expression patterns between genes with different absolute expression values and reduces the impact of inter-sample variation. Data analysis and significance testing were conducted using the Origin 2018 software.

## Results

### Identification and classification of spinach NBS-LRR family members

A total of 114 NBS-LRR family members were identified from the spinach genome (Table 1). Based on the presence of conserved domains, these members were classified into different types: 78 contained the CC domain, 48 contained the LRR domain, one contained the RPW8 domain, and one contained the TIR domain. According to the combination of domains, they were further divided into N (NBS), NL (NBS-LRR), TNL (TIR-NBS-LRR), CN (CC-NBS), CNL (CC-NBS-LRR), and RN (RPW8-NBS) subclasses. Among these, the CN subclass was the most abundant (40 members), followed by the CNL subclass (27 members), N subclass (19 members), and NL subclass (12 members). The TNL and RN subclasses each contained only one member, and other subclasses (*e.g.*, NNL, CNCN) each contained one member.

**Table 1:** Identification and classification of the spinach NBS-LRR family.

Conserved domain	Number	Subclass	Class I	Class II	Number	Total
NBS-ARC	114	N	N	N	19	
				NN	1	20
CC	78		NL	NL	12	
				NNL	1	
LRR	48			NLNL	1	14
		TN	TNL	TNL	1	1
RPW	1	CN	CN	CN	40	
				CNN	1	
TIR	1			CNCN	1	
				NCNN	1	
				NCNCN	1	44
			CNL	CNL	27	
				CNLCN	1	
				CNCNL	2	
				CNLCNL	1	
				CNCNLCN	1	
				NLCL	1	
				NLCN	1	34
		RN	RN	RN	1	1
					114	114

### Chromosomal localization of spinach NBS-LRR genes

**Figure 1:**
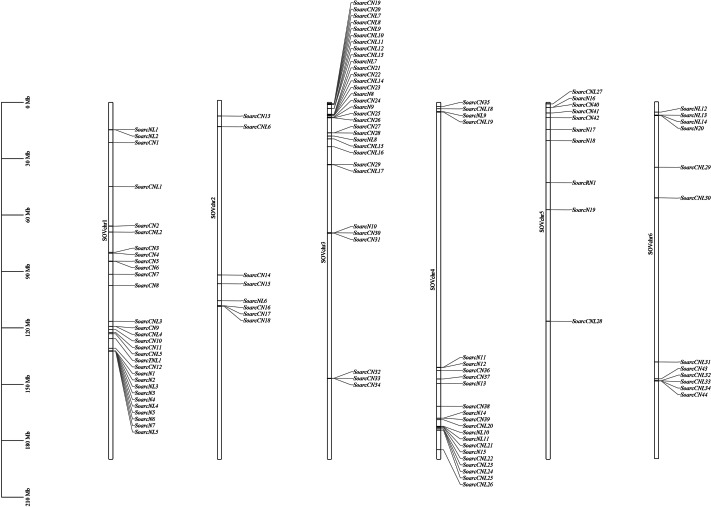
Chromosomal localization of the spinach NBS-LRR family. The vertical axis represents chromosomes, and the horizontal axis represents physical distance (Mb).

Spinach NBS-LRR genes were asymmetrically distributed across the six spinach chromosomes (Fig. 1). Chromosomes 1 and 3 contained the most NBS-LRR genes, with 30 (26.3%) and 32 (28.1%) members, respectively, followed by chromosome 4 (22 genes, 19.3%). Chromosomes 5 and 6 contained 10 and 12 genes, respectively. The distribution of different subclasses varied by chromosome: chromosome 1 was dominated by N and CN subclass genes, chromosome 3 by CN subclass genes, and chromosome 4 by CNL subclass genes. The sole TNL subclass gene was located on chromosome 1, and the sole RN subclass gene was located on chromosome 5.

### Physicochemical properties of spinach NBS-LRR proteins

The 114 spinach NBS-LRR proteins were named based on their domain types and chromosomal locations. The amino acid length of these proteins ranged from 89 to 3,606 aa, with a molecular weight of 10,110.63–411,377.14 D and a theoretical isoelectric point (pI) of 4.68–9.74 (Table S2). Subcellular localization prediction showed that most NBS-LRR proteins were localized in the cytoplasm, while a few were localized in the nucleus or cell membrane.

### Phylogenetic analysis of the spinach NBS-LRR family

**Figure 2:**
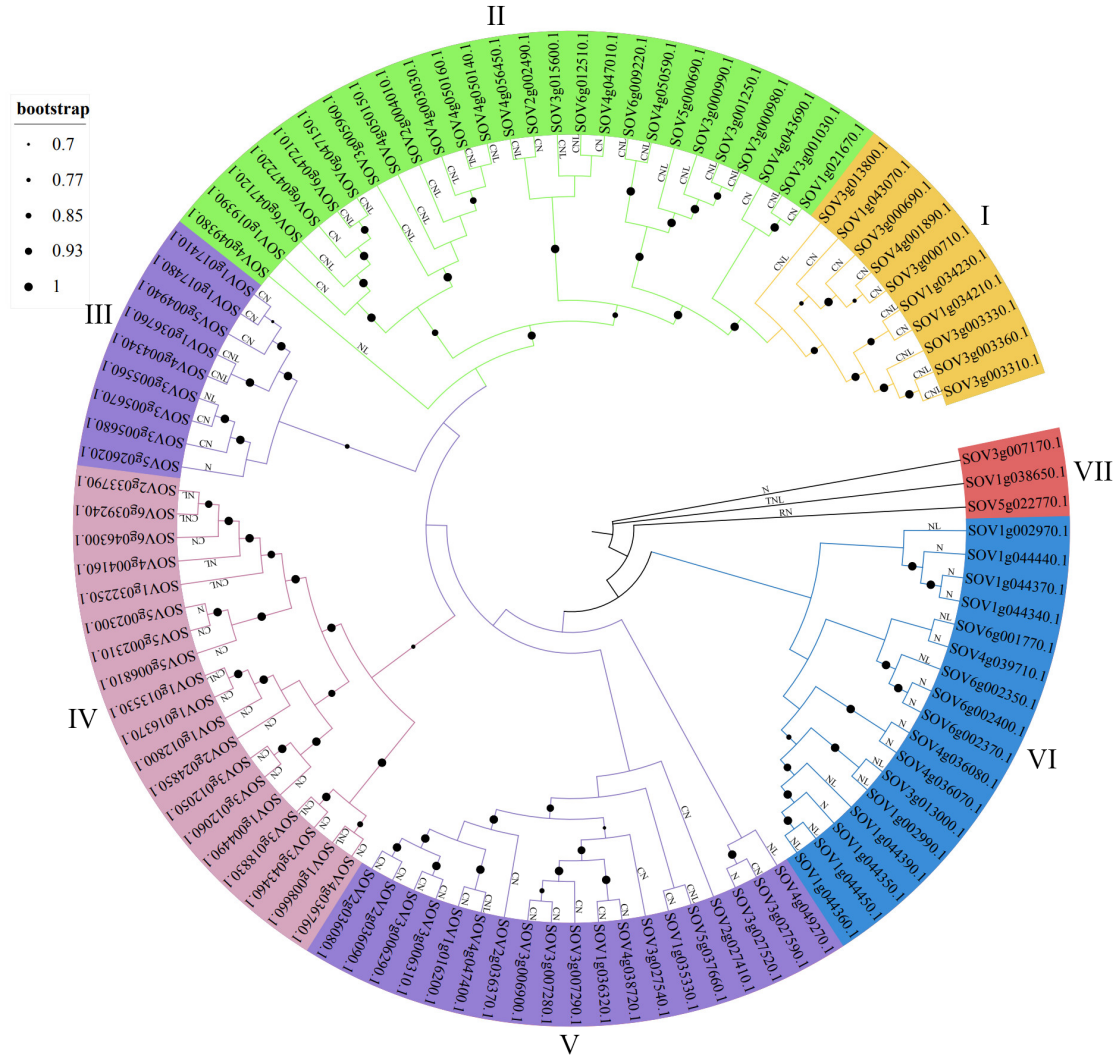
Phylogenetic tree of spinach NBS-LRR family.

After removing sequences with extreme pairwise distance deviations, a phylogenetic tree of 103 NBS-LRR genes was constructed and clustered into eight clades (Clade I–VIII) (Fig. 2). Clade I mainly contained CN and CNL subclass genes, Clade II was dominated by CNL subclass genes, and Clades III–V were enriched in CN subclass genes. Clade VI contained one CN, one N, and one NL subclass gene. Clade VII only contained N and NL subclass genes. Clade VIII contained the only TNL and RN subclass genes, whichwere evolutionarily distant from other sequences. Phylogenetic trees of each domain type showed that sequences within the same domain type had closer evolutionary relationships, indicating evolutionary conservation and similarity among sequences of the same type.

The special difference protein sequences were deleted from the analysis due to the problem of pairwise distance calculation (Fig. 2). The phylogenetic tree of 103 NBS-LRR genes was constructed and clustered into seven groups. There were similarities between sequences of different groups. Clade I, Clade II and Clade III mainly had CN and CNL types; Clade IV and Clade V mainly had CN types; N and NL type sequences were clustered into Clade VI. The only TNL and RN types in the vegetable were clustered into Clade VII with an N type, which had a far evolutionary relationship compared with other sequences. The evolutionary relationship between sequences of each domain category phylogenetic tree was close, and the evolutionary conservation and similarity between sequence types could be seen.

### Analysis of conserved motifs, domains, and gene structures

**Figure 3:**
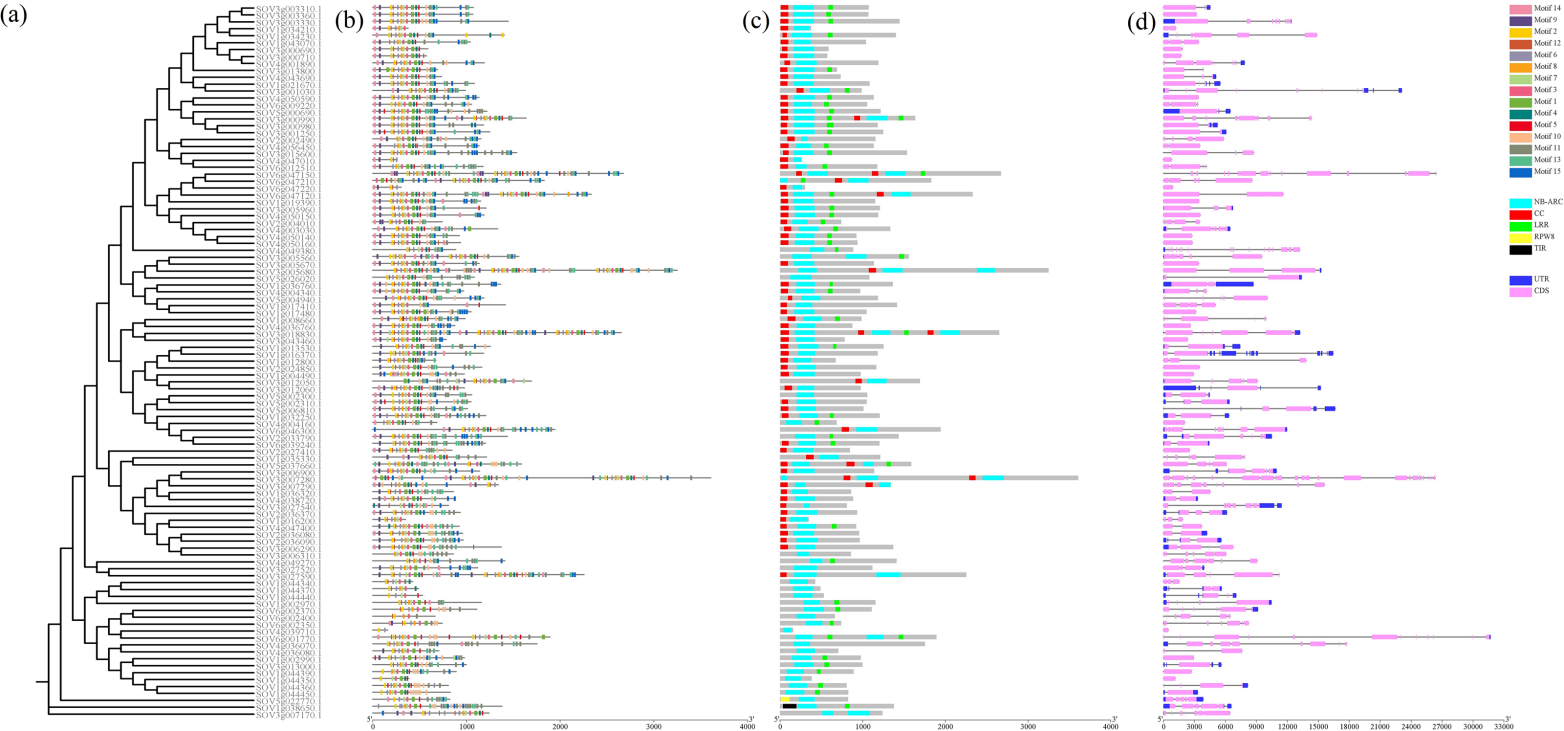
Conserved motifs, domains, and gene structures of the spinach NBS-LRR family. (A) Phylogenetic tree; (B) conserved motif distribution; (C) conserved domain composition; (D) gene structure (exons = pink boxes, introns = black lines, non-coding regions = blue boxes).

Fifteen highly conserved motifs were identified in spinach NBS-LRR proteins using MEME (Figs. 3A and 3B). Motifs 2 and 12 were present in all NBS-LRR proteins, representing the most conserved regions of the family. Genes carrying the CC domain also contained motifs 9 and 14 at conserved positions. Conserved domain analysis showed that Clades I–V mainly contained CC-NBS-LRR domains, while Clades IV–VI lacked LRR domains (Fig. 3C). Clades VI–VIII contained unique NBS-ARC domains, including the only TNL and RN subclass genes. Gene structure analysis showed that most NBS-LRR genes contained multiple exons, introns, and untranslated regions (Fig. 3D). Genes with more conserved motifs had more complex structures. In general, NBS-LRR members from the same phylogenetic branch had similar motif compositions and domain structures, suggesting within-clade functional similarity and between-clade functional diversification.

### Conserved motif structure and multiple sequence alignment

**Figure 4:**
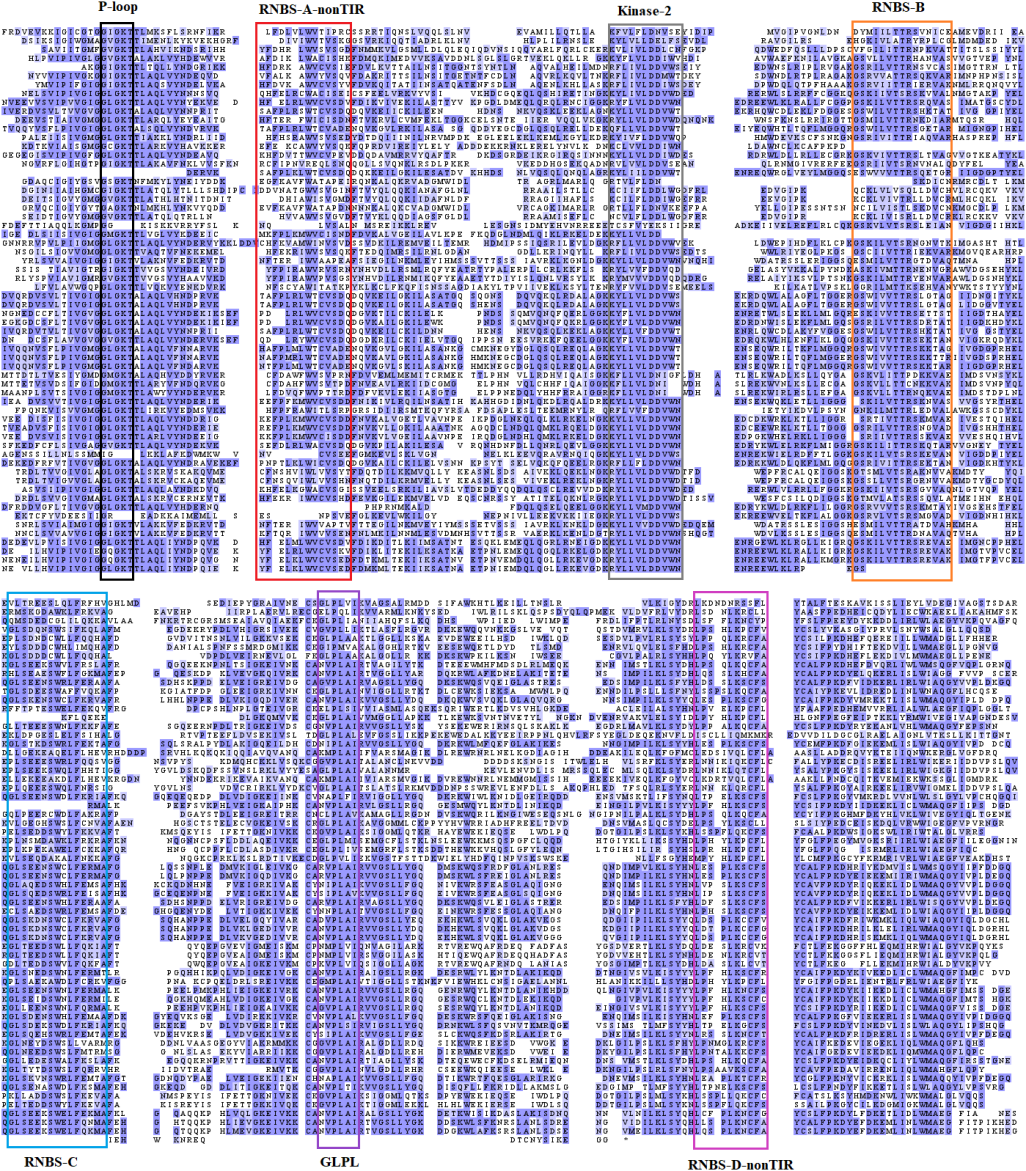
Multiple sequence alignment of conserved motifs in spinach NBS-LRR proteins. Conserved motifs are highlighted in different colors: black, P-loop; red, RNBS-A-nonTIR; grey, Kinase-2; orange, RNBS-B; blue, RNBS-C; purple, GLPL; pink, RNBS-D-nonTIR.

Multiple sequence alignment showed that spinach NBS-LRR proteins contained seven highly conserved motifs: RNBS-D-nonTIR, P-loop, GLPL, Kinase-2, RNBS-C, RNBS-B, and RNBS-A-nonTIR (Fig. 4; Table 2). These motifs displayed conserved alignment and structural features among family members, indicating a common evolutionary origin. Although slight sequence variations existed, the core regions of these motifs were highly conserved, which are likely essential for their functions.

**Table 2:** Structure of conserved motifs of NBS-LRR family in spinach.

Motif No.	Motif sequence	Corresponding domain
motif1	SYYHLPSHLKSCFSYCALFPKDYEIEKEDLIRL	RNBS-D-nonTIR
motif2	IPIVGIGGLGKTTLAQLVYND	p-loop
motif3	GKEIVKKCAGVPLAIRVVGSL	GLPL
motif4	QSLEDAGEEYFSILLRRCFFQ	
motif5	SCKMHDLMHDLAQEV	
motif6	LREELAGKKYLLVLDD	Kinase-2
motif7	TYELQGLSEEDSWELFERVAF	RNBS-C
motif8	GQRGSKILVTTRSEKVARIIG	RNBS-B
motif9	WLEELKDAVYDADDLFDEFVT	
motif10	CLRVLDLSNTGIKSLPDSIG	
motif11	EVLPKSITKLHNLQTLKLRGC	
motif12	FDLRLWVCVSD	RNBS-A-nonTIR
motif13	KLKELPKDJSKLVNLRHLDIS	
motif14	VAQTLLEALQSSEJKEICSJWGYKS	
motif15	CPKLTHMPEGLGKLTSLQTLT	

### Tissue-specific expression of spinach NBS-LRR genes

GWAS studies have identified six downy mildew resistance-related regions on spinach chromosome 3, which encode NBS-LRR proteins and may be involved in spinach downy mildew resistance (She et al., 2018). To explore the expression patterns of *NBS-LRR* genes, 10 candidate genes were selected for qRT-PCR analysis: three from GWAS candidate regions (*SoarcCNL7*, *SoarcCNL8*, *SoarcCN20*), two with special domains (*SoarcRN1*, *SoarcTNL1*), and five from different phylogenetic branches (*SoarcCN4*, *SoarcCN18*, *SoarcCNL20*, *SoarcN19*, *SoarcNL11*).

**Figure 5:**
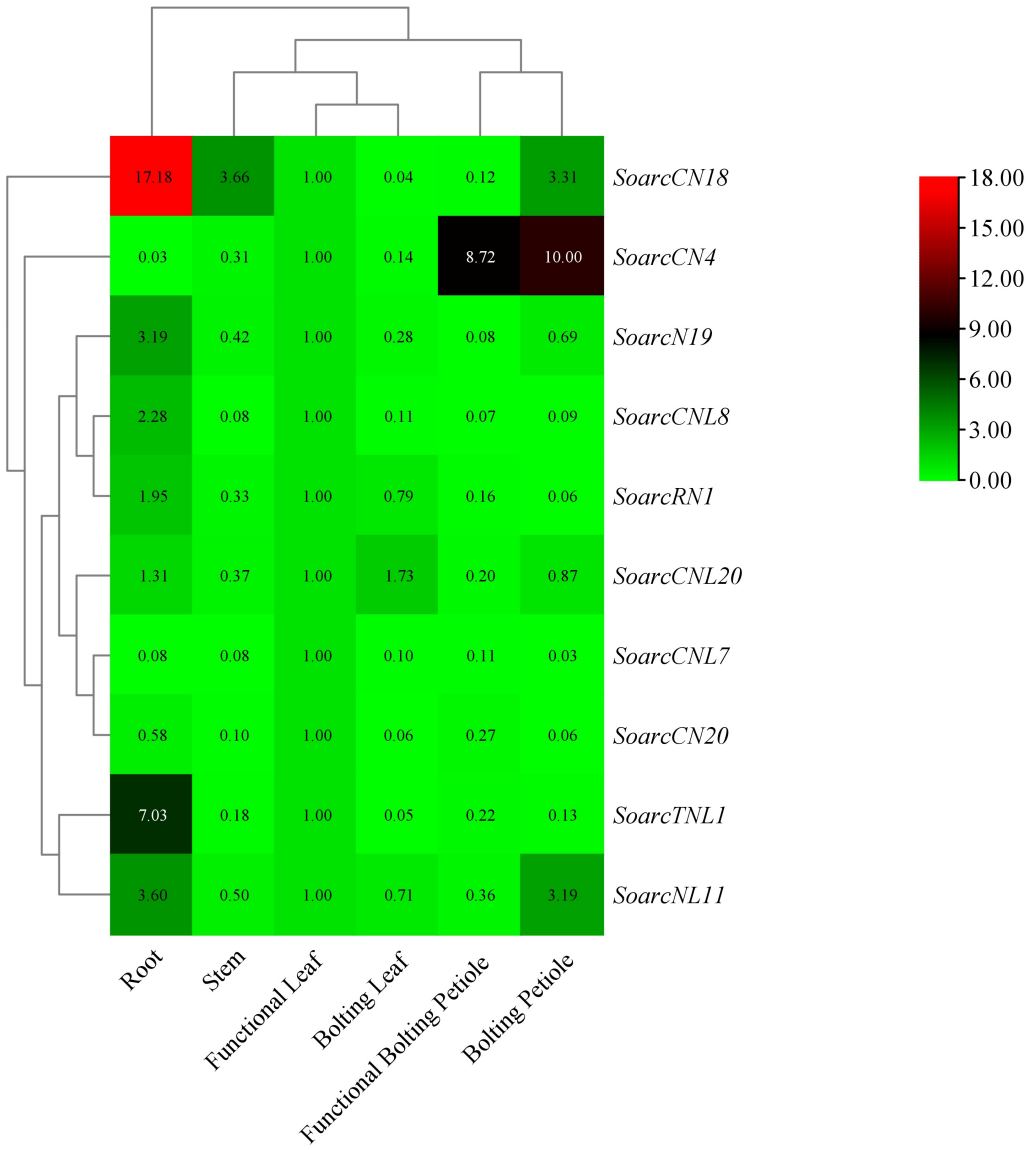
Tissue-specific expression analysis of 10 candidate NBS-LRR genes. The heatmap was generated using relative expression levels. Different colors indicate expression levels: red, high expression; green, low expression.

All candidate genes were expressed in roots, stems, leaves, and petioles, showing tissue-specific expression patterns (Fig. 5). *SoarcCN20* and *SoarcTNL1* were highly expressed in functional petioles, while their expression in functional leaves was relatively low. *SoarcCNL7*, *SoarcCNL8*, and *SoarcRN1* showed higher expression in functional leaves than in functional petioles. *SoarcTNL1* was highly expressed in petioles and roots, and *SoarcRN1* was highly expressed in bolting leaves and stems. Expression-based phylogenetic clustering of expression levels showed that *SoarcCN20* and *SoarcCNL7* were the closest, while *SoarcCN18* was the farthest from other genes. The six tissues were clustered into three subgroups: functional leaves and bolting leaves, functional petioles and bolting petioles, and roots and stems.

### Expression analysis of NBS-LRR genes in resistant and susceptible materials

**Figure 6:**
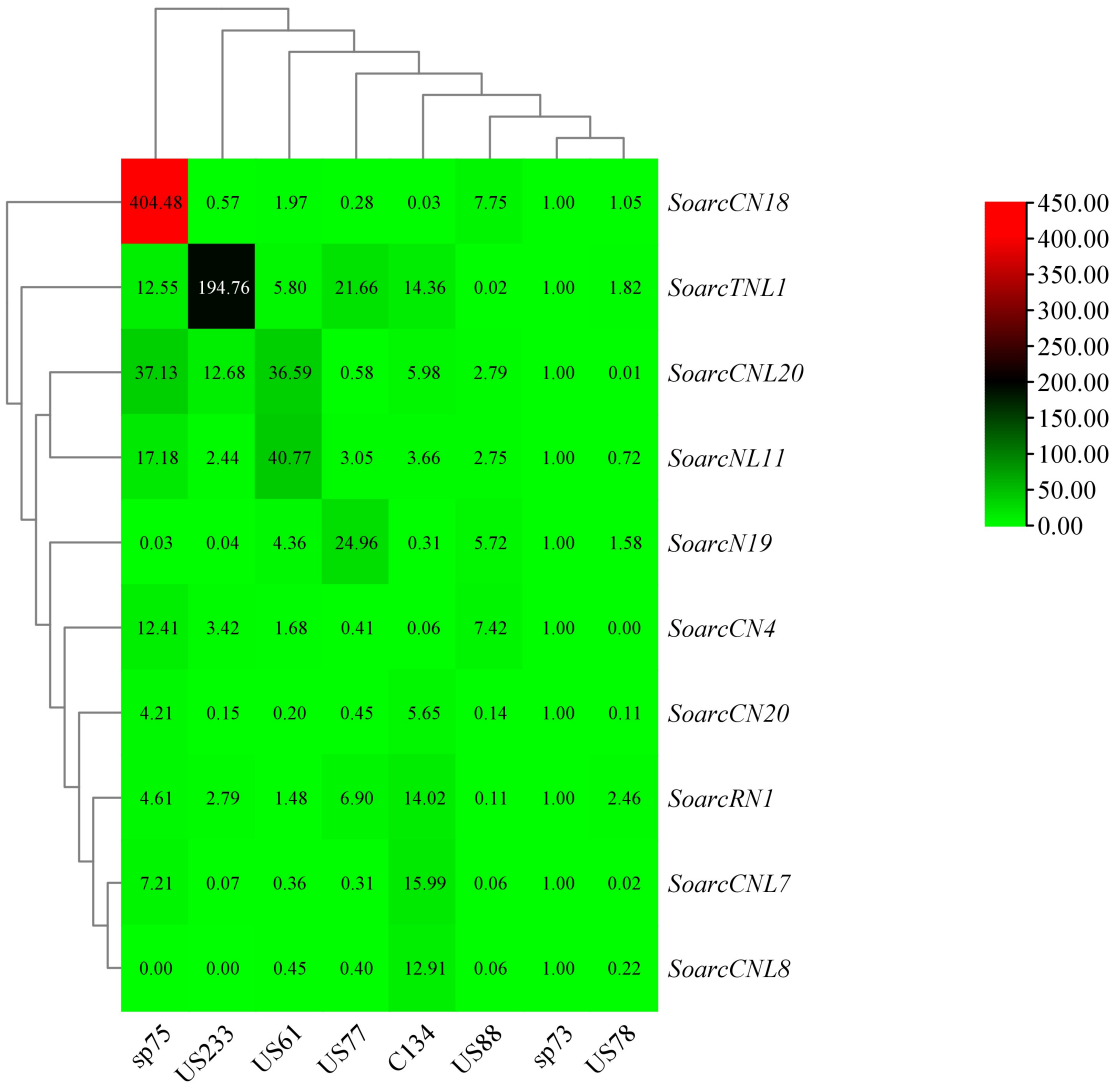
Expression analysis of 10 candidate NBS-LRR genes in resistant and susceptible materials. The heatmap was generated using relative expression levels. Different colors indicate expression levels: red, high expression; green, low expression.

The expression patterns of the 10 candidate genes were analyzed in eight spinach germplasms with different downy mildew resistance levels (Table S3). The results showed that the candidate genes were clustered into two subgroups (Fig. 6). *SoarcCNL20* and *SoarcNL11*, *SoarcCNL7* and *SoarcCNL8* were closely clustered, respectively. *SoarcTNL1* was highly expressed in all materials except the moderately resistant material US88. *SoarcRN1* showed the highest expression in the highly resistant material US78 and was also highly expressed in moderately resistant materials US88 and C134. *SoarcCNL8* had the lowest expression in the susceptible material sp75, and *SoarcCN20* had the lowest expression in the moderately resistant material C134. The three candidate genes (*SoarcCNL7*, *SoarcCNL8*, *SoarcCN20*) showed similar expression patterns in resistant materials compared to susceptible materials. The eight germplasms were clustered into three subgroups: highly resistant materials US77 and US78, moderately resistant materials US61, US88, and C134, and susceptible materials sp73 and sp75.

### Expression analysis of NBS-LRR genes in germplasms with different agronomic traits

**Figure 7:**
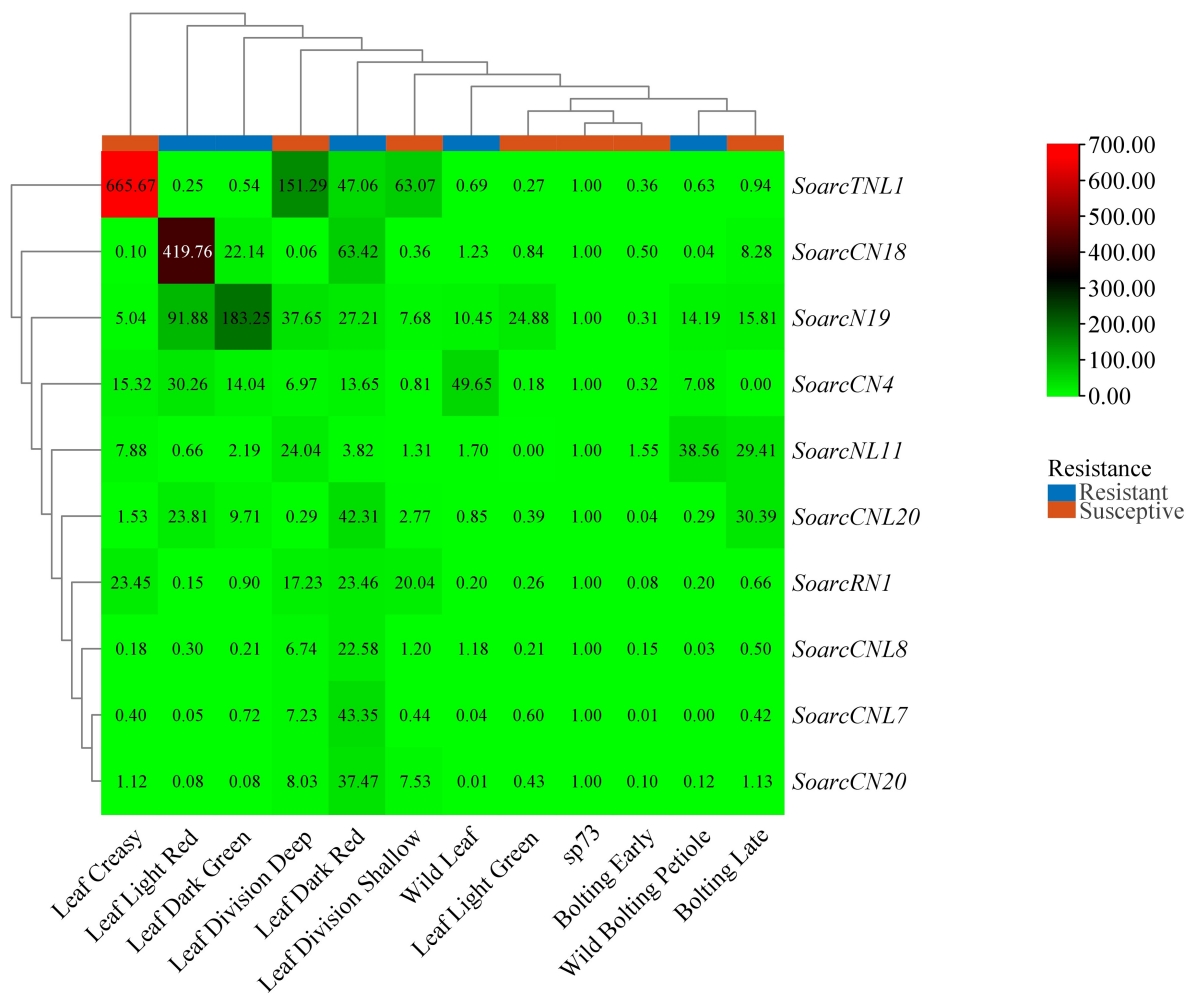
Expression analysis of 10 candidate NBS-LRR genes in germplasms with different agronomic traits. The heatmap was generated using relative expression levels. Different colors indicate expression levels: red, high expression; green, low expression.

The expression patterns of the 10 candidate genes were analyzed in 12 spinach germplasms with different agronomic traits (*e.g.*, leaf color, leaf shape, bolting time) (Table S4). The results showed close clustering of *SoarcCNL8* and *SoarcRN1*, *SoarcCNL7* and *SoarcCN20* were closely clustered, respectively (Fig. 7). *SoarcTNL1* was highly expressed in most traits. *SoarcRN1* showed the lowest expression in light green leaves and the highest expression in dark green leaves, while its expression in dark purple-red leaves was relatively low. *SoarcCNL7* was highly expressed in dark green, light green, and dark purple-red leaves but had low expression in light purple-red leaves. The 12 agronomic traits were clustered into two major clades, each split into two subclades. Each candidate gene displayed unique expression profiles in different traits, reflecting the diversity of trait-related expression patterns.

### Expression dynamics of NBS-LRR genes under downy mildew stress

**Figure 8:**
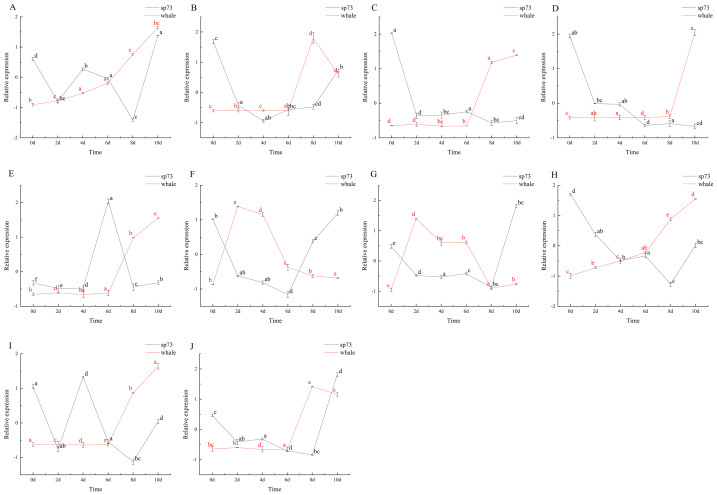
Expression dynamics of 10 candidate NBS-LRR genes under downy mildew stress. (A)–(J) correspond to the 10 candidate genes ((A) *SoarcCN4*; (B) *SoarcCN18*; (C) *SoarcCN20*; (D) *SoarcCNL7*; (E) *SoarcCNL8*; (F) *SoarcCNL20*; (G) *SoarcN19*; (H) *SoarcNL11*; (I) *SoarcRN1*; (J) *SoarcTNL1*). The vertical axis represents relative expression levels (*z*-score normalized). Different letters indicate significant differences at *P* < 0.05. sp73, susceptible material; Whale, resistant material.

To further explore the potential functions of spinach NBS-LRR family genes under downy mildew stress, we analyzed the expression dynamics of 10 candidate genes in downy mildew-susceptible (sp73) and resistant (Whale) spinach materials after pathogen inoculation (Fig. 8).

For the three GWAS-associated NBS-LRR genes: In resistant material (Whale), *SoarcCNL7* showed no significant expression change between 2 and 4 days post-inoculation (dpi); *SoarcCNL8* exhibited stable expression from 0 to 6 dpi followed by a significant increase from 6 to 10 dpi; *SoarcCN20* displayed a similar trend to *SoarcCNL8*. In susceptible material (sp73), *SoarcCNL7* and *SoarcCN20* trended downward; *SoarcCNL8* peaked at 6 dpi (significantly higher than other periods) and remained low at all other time points.

For the remaining seven candidate genes: In resistant material, *SoarcCN4* and *SoarcNL11* showed continuous upregulation from 0 to 10 dpi; *SoarcRN1* and *SoarcTNL1* increased gently from 0 to 6 dpi then rose sharply; *SoarcN19* and *SoarcCNL20* peaked at 2 dpi then decreased. In susceptible material, most candidates decreased from 0 to 2 dpi and increased from 8 to 10 dpi (except *SoarcCNL7*).

Compared with susceptible material, candidates in resistant Whale showed stronger resistance-associated upregulation. Notably, the 10 candidate genes (including three GWAS-associated genes) exhibited inflection points or peaks at 6 and 8 dpi, indicating that candidates in resistant Whale showed stronger resistance-associated upregulation.

### Prediction of the NBS-LRR protein–protein interaction network

**Figure 9:**
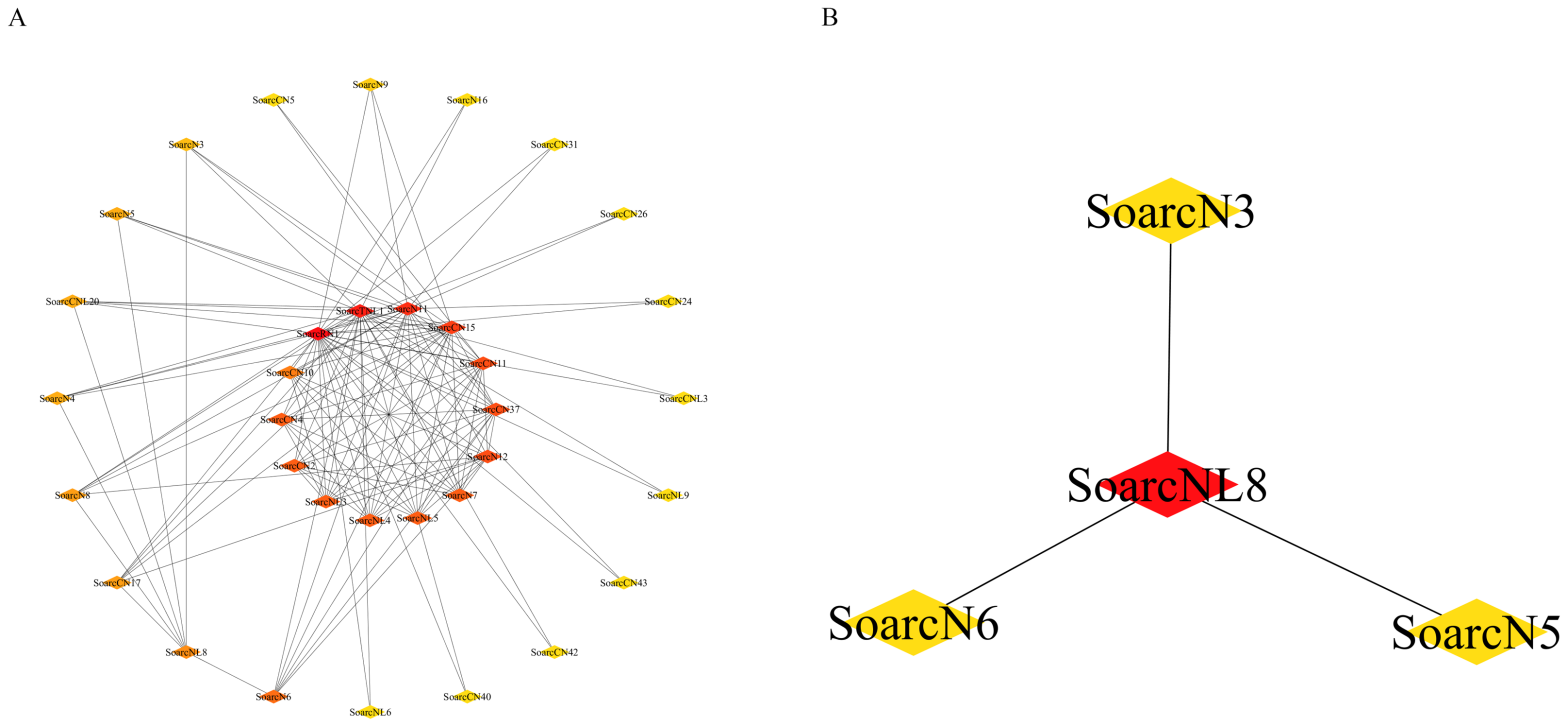
Prediction of the spinach NBS-LRR protein–protein interaction network. (A) Low confidence (0.15); (B) high confidence (0.9). Nodes represent proteins, and lines represent interactions.

PPI network analysis identified 34 interacting proteins at a confidence score threshold of 0.15 (Fig. 9A). The top six proteins with the highest interaction scores were *SoarcRN1*, *SoarcTNL1*, *SoarcN11*, *SoarcCN15*, *SoarcN12*, and *SoarcCN11*, which showed extensive interactions. At a high confidence score threshold of 0.9, four interacting proteins were identified, with *SoarcNL8* as the core protein interacting with *SoarcN3*, *SoarcN5*, and *SoarcN6* (Fig. 9B).

## Discussion

### Identification and characterization of spinach NBS-LRR family genes

In this study, we systematically identified and characterized 114 NBS-LRR family members in spinach, which were classified into CN, CNL, N, NL, TNL, and RN subclasses. The CN subclass (40 members) was the most abundant, followed by the CNL subclass (27 members), which is consistent with the distribution pattern of NBS-LRR subclasses in other dicotyledonous plants such as Arabidopsis (Shao et al., 2016) and tomato (Sagi, Deokar & Tar’an, 2017). The scarcity of TNL and RN subclasses (one member each) in spinach may reflect species-specific evolutionary adaptations, as different plant species have evolved distinct NLR repertoires in response to their unique pathogen pressures (Takken & Goverse, 2012). Notably, our subclass classification and systematic characterization complement the recent pan-NLRome analysis by She et al. (2026), which focused on the discovery of resistance loci but did not provide detailed insights into the structural and evolutionary features of the spinach NBS-LRR family. For example, She et al. (2026) identified candidate resistance genes but did not analyze their chromosomal distribution, conserved motifs, or phylogenetic relationships, which are critical for understanding their functional diversity.

The asymmetric chromosomal distribution of spinach NBS-LRR genes, with enrichment on chromosomes 1 and 3 (26.3% and 28.1%, respectively), is consistent with the findings in other crops such as rice and wheat, where NBS-LRR genes often cluster in specific chromosomal regions (Sukarta, Slootweg & Goverse, 2016). This clustering may be the result of tandem duplication and segmental duplication events, which are key drivers of NBS-LRR gene family expansion (Innes, 2004). Chromosome 3, which harbors the highest number of NBS-LRR genes (32 members), has been previously linked to downy mildew resistance loci in spinach (She et al., 2018; She et al., 2026), suggesting that this chromosomal region may be a hot spot for resistance gene evolution. The conserved motifs (motifs 2 and 12) identified in all NBS-LRR proteins are essential for ATP/GTP binding and hydrolysis (Innes, 2004), and their high conservation across the family supports a common evolutionary origin of spinach NBS-LRR genes.

### Tissue-specific and trait-related expression of NBS-LRR genes

Spinach germplasms (cultivated and wild) exhibit rich morphological diversity, including variation in traits such as carotenoid content, folate content, oxalate content, and nitrate content (Roberts & Moreau, 2016). Human selection has driven convergent evolution of crop morphological traits, such as expanded edible parts and reduced undesirable flavors (Rendón-Anaya & Herrera-Estrella, 2018; Ahmad et al., 2019). Spinach has a short cultivation history, and there are few genetic and morphological differences between cultivated and wild species (Ribera et al., 2020). Selective sweep analysis of spinach genomes has identified Quantitative Trait Loci (QTL) and GWAS signals associated with important traits such as flowering time, bolting time, plant type, leaf surface texture, leaf base shape, and petiole color and width (Cai et al., 2021). The crinkled leaf trait may be a result of human selection (Cai et al., 2021).

In this study, the 10 candidate NBS-LRR genes were broadly expressed across spinach tissues with distinct specificity. For example, *SoarcTNL1* was highly expressed in petioles and roots, and *SoarcRN1* was highly expressed in bolting leaves and stems, suggesting tissue-specific functional roles. Additionally, the candidate genes showed differential expression in germplasms with different downy mildew resistance levels and agronomic traits, indicating that *NBS-LRR* genes may be involved in the regulation of multiple traits. The clustering of resistant and susceptible materials based on gene expression levels suggests that the expression patterns of *NBS-LRR* genes can be used to distinguish between resistant and susceptible materials. Further studies are needed to verify direct links between *NBS-LRR* gene expression and target traits.

### Response of NBS-LRR genes to downy mildew stress

In spinach research and breeding, abiotic stress tolerance and quality-related traits have long received relatively limited attention, whereas disease resistance—particularly against downy mildew—has consistently been a primary goal (Hirakawa et al., 2021). When colonizing host plants, most downy mildew pathogens induce distinct foliar signs and symptoms; consequently, the diagnosis of downy mildew heavily depends on the direct observation of these symptoms and signs *via* visible light microscopy (Salcedo et al., 2020). Downy mildew encompasses a broad spectrum of specialized pathogens, which can inflict severe damage to plants in commercial production systems, landscape settings, and natural ecosystems (Gascuel et al., 2015).

Traditional pathogen inoculation mainly uses spray methods (She et al., 2018; Kandel et al., 2020), which require precise adjustment of spore concentration and plant pretreatment; environmental factors (temperature, light, humidity) strongly determine infection success. In the present study, we used a refined inoculation method: a high-concentration pathogen suspension was first purified in the laboratory, followed by spot inoculation of spinach leaves. To enhance the specificity of the plant–pathogen interaction, no dark treatment was applied, and indoor humidity was maintained consistently throughout the experiment. During the inoculation period, pathogen sporulation (visible breakout) was not observed until the 9th day post-inoculation (dpi); however, typical downy mildew-related symptoms, including leaf yellowing, curling, and growth inhibition, had already appeared within the first 8 days.

Analysis of candidate gene expression patterns revealed distinct dynamics in susceptible material: most candidate genes exhibited a downward expression trend from 0 to 2 dpi, whereas a general upward trend was observed from 8 to 10 dpi. In contrast, in resistant materials, the candidate genes showed more pronounced upregulation associated with resistance activation, along with potential resistance-related mechanisms. Importantly, inflection points and expression peaks of the candidate genes were detected at 6 dpi and 8 dpi post-inoculation with downy mildew pathogens, which mark key time points for spinach defense activation against downy mildew. These results are consistent with previous studies showing that NBS-LRR genes are rapidly induced by pathogen infection (Kandel et al., 2020). For example, *SoarcCNL8* and *SoarcCN20* in resistant material showed a significant increase in expression from 6 to 10 dpi, suggesting that they may play late-stage defense functions. These findings provide a valuable framework for dissecting the molecular mechanisms underlying spinach’s response to downy mildew infection.

It is important to note that the observed differential expression of NBS-LRR candidate genes between resistant and susceptible spinach materials, and their upregulation under downy mildew stress, only indicates an association between these genes and downy mildew resistance, not a direct causal relationship. Functional validation experiments (*e.g.*, CRISPR/Cas9-mediated gene knockout, overexpression of candidate genes in susceptible spinach genotypes, and complementation tests) are required to confirm the direct functional roles of these NBS-LRR genes in spinach downy mildew resistance. The expression dynamics and PPI network results reported in this study provide a prioritized list of candidate genes for subsequent functional validation, which will accelerate the dissection of the molecular mechanisms underlying spinach defense against *P. effusa*.

## Conclusions

In this study, 114 NBS-LRR family members were identified from the spinach genome using bioinformatics methods, and classified into subclasses dominated by CN and CNL based on conserved domains. These genes were asymmetrically distributed across spinach chromosomes, with the majority located on chromosomes 1 and 3. The NBS-LRR proteins had diverse physicochemical properties but shared conserved motifs (notably motifs 2 and 12), showing evolutionary conservation within phylogenetic clades and functional diversification between clades.

The 10 candidate NBS-LRR genes were widely expressed in different spinach tissues (roots, stems, leaves, petioles) with tissue-specific patterns. They showed differential expression in downy mildew-resistant and susceptible materials, and distinct expression profiles in germplasms with different agronomic traits. Under downy mildew stress, the candidate genes in the resistant material (Whale) showed stronger resistance-associated upregulation compared to the susceptible material (sp73), with inflection points and peaks at 6 dpi and 8 dpi. PPI analysis identified core resistance proteins *SoarcRN1* and *SoarcNL8*. Future research will focus on verifying the causal roles of these candidate genes in downy mildew resistance using reverse genetic approaches, and exploring their molecular mechanisms of action in the spinach innate immune response to *P. effusa*.

### Data availability compliance

All data generated or analyzed during this study are included in this published article and its supplementary information files (Tables S1–S4, Figs. 1–9). The spinach genome data used in this study are publicly available from Spinachbase (http://spinachbase.org/ftp/genome/Monoe-Viroflay/).

## Supplemental Information

10.7717/peerj.21599/supp-1 Table S1 PCR primer information used in this study;

10.7717/peerj.21599/supp-2 Table S2 Physicochemical properties of spinach NBS-LRR family proteins;

10.7717/peerj.21599/supp-3 Table S3 Information on spinach germplasms with different downy mildew resistance levels

10.7717/peerj.21599/supp-4 Table S4 Information on spinach germplasms with different agronomic traits

10.7717/peerj.21599/supp-5 Supplemental Information 5 MIQE checklist

10.7717/peerj.21599/supp-6 Data S1 Protein sequence information and Original data values

## Additional Information and Declarations

### Data Availability

The following information was supplied regarding data availability: The data is available in the Supplemental Files.
