# Supplementary material for: Genome-wide identification and expression analysis of nucleotide-binding site leucine-rich repeat genes associated with downy mildew resistance in spinach
Source: PeerJ. 2026 Aug 14;14:e21599. doi: 10.7717/peerj.21599 (PMC13480143; doi:10.7717/peerj.21599)
Supplement: Supplementary file 1 [file peerj-14-21599-s001.pdf]

Table S1: PCR primer information used in this study.

Locus	Primer	Sequence (5′→3′)	temperature
SoarcCNL7	F	ACGCTTGGGGCTACAAAACT	60.18
	R	CTAGGGGAAAGCTCTCGCTG	
SoarcCNL8	F	GGTCAAGGGGGAAGCAAGAT	59.67
	R	TAAGCGCCATGAACTCTCGT	
SoarcCN20	F	TGATCTTCAACGCACCGTCT	60.11
	R	TAAGCTCCTCCAGCCAATGC	
SoarcRN1	F	GACCTGGTGAAGTTGCCTGA	60.11
	R	GGGCAAGCATACACCCTCAT	
SoarcTNL1	F	CTATGACGTCGACCCGTCTG	59.97
	R	CTGGCAACCTCGTCTTCACT	
SoarcCN4	F	TGACCATCTTCGGGTTTGGG	59.96
	R	ACGCGCCACTCTTTATCACA	
SoarcCN18	F	CAAAGACAAACGCGGGATGG	60.03
	R	CCCGGGTCTACCTTGAGAGA	
SoarcCNL20	F	TAGCGCACTGCCACAACTTA	59.97
	R	TGTTGACCTTATTGGCCGCT	
SoarcN19	F	TGGTTGCCCGAAGTTGAAGT	60.11
	R	GTGCTCCAGCAGTAACCCAT	
SoarcNL11	F	TCGAGTGGGCTGCTTTCATT	60.04
	R	CCGTGTAGACCCCAGCAAAT	
