# Supplementary material for: Genome-wide identification and expression analysis of nucleotide-binding site leucine-rich repeat genes associated with downy mildew resistance in spinach
Source: PeerJ. 2026 Aug 14;14:e21599. doi: 10.7717/peerj.21599 (PMC13480143; doi:10.7717/peerj.21599)
Supplement: Supplementary file 2 [file peerj-14-21599-s002.pdf]

Table S2 Physicochemical properties of NBS-LRR family of spinach

Gene ID	Rename	Aminoacid number(aa)	Molecular weight (D)	Theoretical isoelectric point(PI)	Instability index	Aliphatic index	GRAVY	Subcellular localization
SOV1g002970.1	*SoarcNL1*	1155	130540.1	6.32	44.49	94.91	-0.224	Cytoplasm
SOV1g002990.1	*SoarcNL2*	977	112334.5	6.3	41.26	109.23	-0.053	Cytoplasm
SOV1g004490.1	*SoarcCN1*	975	111314.3	5.79	50.44	98.99	-0.136	Cytoplasm
SOV1g008660.1	*SoarcCNL1*	985	111147.6	8.43	35.19	109.34	0.056	Cytoplasm
SOV1g012800.1	*SoarcCN2*	673	76331.04	5.75	41.89	103.27	-0.181	Cytoplasm
SOV1g013530.1	*SoarcCNL2*	1252	142050.1	5.84	43.37	96.35	-0.205	Cytoplasm
SOV1g016200.1	*SoarcCN3*	348	40224.22	9.27	46.38	95.78	-0.214	Cytoplasm
SOV1g016370.1	*SoarcCN4*	1181	133923.1	5.77	46.23	103.9	-0.037	Cytoplasm
SOV1g017410.1	*SoarcCN5*	1415	157424.9	6.01	45.27	102.94	-0.004	Cytomembrane、Nucleus
SOV1g017480.1	*SoarcCN6*	1047	119617.6	8.09	52.11	98.3	0.186	Cytoplasm
SOV1g019390.1	*SoarcCN7*	1151	131246.3	8.28	49.88	104.1	0.125	Cytoplasm
SOV1g021670.1	*SoarcCN8*	1083	121770.2	5.96	45.89	101.07	0.154	Cytoplasm
SOV1g032250.1	*SoarcCNL3*	1206	137386.3	8.46	47.74	102.27	-0.2	Cytoplasm
SOV1g034210.1	*SoarcCN9*	377	42401.11	8.72	26.68	98.78	-0.163	Cytoplasm
SOV1g034230.1	*SoarcCNL4*	1401	159162	6.1	43.38	90.54	-0.202	Cytoplasm、Cytomembrane、Nucleus
SOV1g035330.1	*SoarcCN10*	1213	138133.3	8.37	42.04	94.01	0.211	Cytoplasm
SOV1g036320.1	*SoarcCN11*	859	98156.3	8.51	28.77	93.91	-0.288	Cytoplasm
SOV1g036760.1	*SoarcCNL5*	1364	156040.8	5.51	49.27	98.83	-0.301	Cytoplasm
SOV1g038650.1	*SoarcTNL1*	1378	155434	6.17	44.49	102.15	-0.213	Cytomembrane
SOV1g043070.1	*SoarcCN12*	1040	118597.3	6.04	41.51	94.75	-0.265	Cytoplasm
SOV1g044340.1	*SoarcN1*	426	49834.41	8.51	36.63	82.65	-0.63	Cytoplasm
SOV1g044350.1	*SoarcN2*	384	44002.27	8.4	42.81	109.32	-0.123	Cytoplasm
SOV1g044360.1	*SoarcNL3*	805	91374.25	6.81	46.5	109.42	-0.067	Cytoplasm
SOV1g044370.1	*SoarcN3*	489	55983.78	6.55	32.67	87.28	-0.408	Cytoplasm

Table S2 Physicochemical properties of NBS-LRR family of spinach

Gene ID	Rename	Aminoacid number(aa)	Molecular weight (D)	Theoretical isoelectric point(PI)	Instability index	Aliphatic index	GRAVY	Subcellular localization
SOV1g044380.1	*SoarcN4*	409	45405.39	5.77	38.2	101.05	-0.056	Cytoplasm
SOV1g044390.1	*SoarcNL4*	890	100737.8	7.68	48.74	102.46	-0.118	Cytoplasm、Cytomembrane
SOV1g044410.1	*SoarcN5*	368	41722.26	5.87	38.99	106.66	-0.102	Cytoplasm
SOV1g044420.1	*SoarcN6*	401	45697.08	8.06	38.89	93.57	-0.179	Cytoplasm
SOV1g044440.1	*SoarcN7*	529	60226.67	7.87	40.39	87.71	-0.384	Cytoplasm
SOV1g044450.1	*SoarcNL5*	826	94004.57	5.95	45.8	106.61	-0.071	Cytoplasm、Cytomembrane
SOV2g002490.1	*SoarcCN13*	1154	130643.7	5.99	45.69	101.07	-0.109	Cytoplasm、Cytomembrane
SOV2g004010.1	*SoarcCNL6*	740	84793.61	5.94	45.22	97.95	-0.252	Cytoplasm
SOV2g024850.1	*SoarcCN14*	1164	133375.2	6.57	51.81	99.57	-0.293	Cytoplasm、Cytomembrane
SOV2g027410.1	*SoarcCN15*	845	95312.03	7.01	38.11	98.21	-0.182	Cytoplasm
SOV2g033790.1	*SoarcNL6*	1435	162937	5.92	51.88	102.4	-0.106	Cytoplasm
SOV2g036080.1	*SoarcCN16*	958	110103.2	7.58	42.64	101.41	-0.247	Cytoplasm
SOV2g036090.1	*SoarcCN17*	964	110394.7	8.16	43.93	104.84	-0.209	Cytoplasm
SOV2g036370.1	*SoarcCN18*	933	107203.9	8.9	37.52	95.64	-0.337	Cytoplasm
SOV3g000690.1	*SoarcCN19*	587	65933.78	7.89	45.27	95.84	-0.205	Cytoplasm
SOV3g000710.1	*SoarcCN20*	572	64140.58	5.43	42.54	95.45	-0.187	Cytoplasm
SOV3g000980.1	*SoarcCNL7*	1181	134509.3	5.74	40.38	96.29	-0.304	Cytoplasm
SOV3g000990.1	*SoarcCNL8*	1635	187026.1	5.76	43.33	99.77	-0.213	Cytoplasm
SOV3g001030.1	*SoarcCNL9*	987	110944	8.57	37.5	93.64	-0.177	Cytoplasm、Nucleus
SOV3g001250.1	*SoarcCNL10*	1248	141869.9	5.81	45.29	94.08	-0.323	Cytoplasm
SOV3g003310.1	*SoarcCNL11*	1072	120603.5	8.63	36.55	99.56	-0.096	Cytoplasm
SOV3g003330.1	*SoarcCNL12*	1445	164457.6	6.36	37.91	100.35	-0.183	Cytoplasm、Cytomembrane
SOV3g003360.1	*SoarcCNL13*	1069	121223.9	7.83	41.44	100.2	-0.134	Cytoplasm
SOV3g005560.1	*SoarcNL7*	1559	179231.9	5.86	41.72	95.47	-0.219	Cytoplasm

Table S2 Physicochemical properties of NBS-LRR family of spinach

Gene ID	Rename	Aminoacid number(aa)	Molecular weight (D)	Theoretical isoelectric point(PI)	Instability index	Aliphatic index	GRAVY	Subcellular localization
SOV3g005670.1	*SoarcCN21*	1136	129013.2	7.95	44.23	98.15	-0.183	Cytoplasm
SOV3g005680.1	*SoarcCN22*	3248	369674.6	7.21	40.7	98.79	-0.209	/
SOV3g005960.1	*SoarcCNL14*	1208	138134.6	7.23	44.42	100.09	-0.265	Cytomembrane
SOV3g006290.1	*SoarcCN23*	1370	157825.2	8.5	48.41	101.77	-0.126	Cytoplasm
SOV3g006310.1	*SoarcN8*	858	96670.97	8.89	50.09	101.66	-0.123	Cytoplasm
SOV3g006900.1	*SoarcCN24*	1139	129062.4	8.11	40.62	99.74	-0.131	Cytoplasm
SOV3g007170.1	*SoarcN9*	1239	141099.2	5.79	47.73	94.33	-0.4	Cytoplasm
SOV3g007280.1	*SoarcCN25*	3606	411377.1	8.19	43.53	98.5	-0.149	/
SOV3g007290.1	*SoarcCN26*	1339	152729.7	9.12	44.73	102.41	-0.173	Cytoplasm、Nucleus
SOV3g012050.1	*SoarcCN27*	1692	190650.9	6.59	41.61	95.37	-0.156	Cytoplasm
SOV3g012060.1	*SoarcCN28*	976	111293.9	6.19	46.84	96.75	-0.167	Cytoplasm
SOV3g013000.1	*SoarcNL8*	998	113629.3	6.8	39.73	98.61	-0.246	Cytoplasm
SOV3g013800.1	*SoarcCNL15*	690	78159.29	6.43	42.23	103.12	-0.17	Cytoplasm
SOV3g015600.1	*SoarcCNL16*	1536	174635.9	7.54	47.77	97.02	-0.076	Cytoplasm
SOV3g018760.1	*SoarcCN29*	332	38193.91	8.18	47.33	95.96	-0.126	Cytoplasm、Nucleus
SOV3g018830.1	*SoarcCNL17*	2651	302821.6	7.27	45.15	106.7	-0.064	Cytoplasm
SOV3g027520.1	*SoarcN10*	1118	126720.1	8.89	48.73	97.5	-0.336	Cytoplasm
SOV3g027540.1	*SoarcCN30*	808	93213.84	8.87	45.6	89.89	-0.436	Cytoplasm
SOV3g027590.1	*SoarcCN31*	2253	256659.7	8.31	44.11	97.87	-0.333	Cytoplasm
SOV3g043460.1	*SoarcCN32*	781	90363.1	8.51	42.85	104.16	-0.12	Cytoplasm
SOV3g043470.1	*SoarcCN33*	213	24792.55	5.98	36.77	111.55	-0.142	Cytoplasm
SOV3g043500.1	*SoarcCN34*	168	19003.13	9.12	33.14	115.83	0.01	Cytoplasm
SOV4g001890.1	*SoarcCN35*	1189	134056.2	5.68	49.51	96.43	-0.193	Cytoplasm
SOV4g003030.1	*SoarcCNL18*	1333	152325.3	6.46	47.36	94.31	-0.302	Cytoplasm、Cytomembrane、Nucleus

Table S2 Physicochemical properties of NBS-LRR family of spinach

Gene ID	Rename	Aminoacid number(aa)	Molecular weight (D)	Theoretical isoelectric point(PI)	Instability index	Aliphatic index	GRAVY	Subcellular localization
SOV4g004160.1	*SoarcNL9*	685	78021.28	5.71	42.26	107.15	-0.042	Cytoplasm
SOV4g004340.1	*SoarcCNL19*	970	110250.3	6.14	43.04	102.22	-0.206	Cytoplasm
SOV4g036070.1	*SoarcN11*	1753	200806.7	5.63	50.04	98.17	-0.152	Nucleus
SOV4g036080.1	*SoarcN12*	705	79338.2	6.46	43.78	102.98	-0.098	Cytoplasm
SOV4g036760.1	*SoarcCN36*	875	99400.34	6.23	50.37	105.46	-0.122	Cytoplasm
SOV4g038720.1	*SoarcCN37*	884	100788.3	8.04	37.25	105.81	-0.146	Cytoplasm
SOV4g039710.1	*SoarcN13*	158	18012.08	9.63	38.02	105.51	-0.162	Cytoplasm、Nucleus
SOV4g043690.1	*SoarcCN38*	733	83401.04	6.47	40.53	94.53	-0.197	Cytoplasm、Cytomembrane、Nucleus
SOV4g047000.1	*SoarcN14*	509	58410.06	6.83	44.12	104.34	-0.125	Cytoplasm
SOV4g047010.1	*SoarcCN39*	262	29329.66	6.04	30.1	96.03	-0.235	Cytoplasm
SOV4g047400.1	*SoarcCNL20*	921	106558.6	8.37	47.92	105.5	-0.249	Cytoplasm
SOV4g049270.1	*SoarcNL10*	1410	160949	5.4	50.1	92.86	-0.295	Cytoplasm
SOV4g049380.1	*SoarcNL11*	886	99681.6	8.31	39.56	105.05	0.037	Golgi apparatus
SOV4g049510.1	*SoarcCNL21*	1744	201085	5.89	45.65	97.32	-0.29	Cytoplasm、Cytomembrane
SOV4g049650.1	*SoarcN15*	89	10110.63	4.68	16.24	121.46	0.071	Cytoplasm
SOV4g050140.1	*SoarcCNL22*	924	105791.3	8.11	40.42	98.28	-0.267	Cytoplasm
SOV4g050150.1	*SoarcCNL23*	1187	136514	6.31	49.48	97.43	-0.314	Cytoplasm
SOV4g050160.1	*SoarcCNL24*	936	106635	7.56	43.65	100.74	-0.224	Cytoplasm
SOV4g050590.1	*SoarcCNL25*	1133	129471.4	8.12	47.42	96.24	-0.237	Cytoplasm
SOV4g056450.1	*SoarcCNL26*	1133	128604.9	7.62	48.06	96.23	-0.155	Cytoplasm
SOV5g000690.1	*SoarcCNL27*	1217	139564.9	5.32	49.46	93.93	-0.359	Cytoplasm、Nucleus
SOV5g002300.1	*SoarcN16*	1055	119694.7	8.59	46.02	105.86	-0.118	Cytoplasm、Cytomembrane
SOV5g002310.1	*SoarcCN40*	1048	119610.7	7.84	44.46	103.95	-0.159	Cytoplasm
SOV5g004940.1	*SoarcCN41*	1185	134841.1	6	48.91	103.23	-0.144	Cytoplasm

Table S2 Physicochemical properties of NBS-LRR family of spinach

Gene ID	Rename	Aminoacid number(aa)	Molecular weight (D)	Theoretical isoelectric point(PI)	Instability index	Aliphatic index	GRAVY	Subcellular localization
SOV5g006810.1	*SoarcCN42*	1010	115262.2	8.74	44.35	101.19	-0.107	Cytoplasm
SOV5g011180.1	*SoarcN17*	275	30994.35	6.14	33.23	98.11	-0.323	Cytoplasm
SOV5g014240.1	*SoarcN18*	388	43301.12	5.19	41.38	90.41	-0.303	Cytoplasm
SOV5g022770.1	*SoarcRN1*	823	92799.14	6.3	47.38	107.64	0.014	Cytoplasm
SOV5g026020.1	*SoarcN19*	1081	123276.8	5.71	43.68	103.75	-0.229	Cytoplasm
SOV5g037660.1	*SoarcCNL28*	1586	182575.4	8.72	40.59	95.22	-0.403	Cytoplasm
SOV6g001770.1	*SoarcNL12*	1892	212298.9	6.26	43.09	99.01	-0.152	Cytoplasm、Nucleus
SOV6g002350.1	*SoarcNL13*	740	84756.85	8.9	40.9	90.39	-0.357	Cytoplasm、Cytomembrane、Nucleus
SOV6g002370.1	*SoarcNL14*	1109	125936.8	7.59	45.32	95.36	-0.157	Cytoplasm
SOV6g002400.1	*SoarcN20*	663	75463.14	7.71	37.78	97.93	-0.29	Cytoplasm
SOV6g009220.1	*SoarcCNL29*	1054	120099.1	6.13	39.88	97.18	-0.191	Cytoplasm
SOV6g012510.1	*SoarcCNL30*	1176	134281.7	6.21	47.28	99.86	-0.154	Cytoplasm
SOV6g039240.1	*SoarcCNL31*	1203	136759.2	6.74	47.39	107.64	-0.103	Cytoplasm、Cytomembrane
SOV6g046300.1	*SoarcCN43*	1942	218754.3	6.92	44.39	99.39	-0.085	Cytoplasm
SOV6g047120.1	*SoarcCNL32*	2330	264357.1	8.22	40.97	102.67	-0.154	Cytoplasm
SOV6g047150.1	*SoarcCNL33*	2672	303538.1	8.21	44.22	97.23	-0.207	Cytoplasm
SOV6g047210.1	*SoarcCNL34*	1829	208489.2	6.54	47.6	99.34	-0.218	Cytoplasm、Cytomembrane
SOV6g047220.1	*SoarcCN44*	302	34452	4.82	39.68	95.23	-0.392	Cytoplasm
