# Supplementary material for: Genome-wide identification and expression analysis of nucleotide-binding site leucine-rich repeat genes associated with downy mildew resistance in spinach
Source: PeerJ. 2026 Aug 14;14:e21599. doi: 10.7717/peerj.21599 (PMC13480143; doi:10.7717/peerj.21599)
Supplement: Supplementary file 3 [file peerj-14-21599-s003.pdf]

Table S3 Information on spinach resistant materials

Name	Resistance to downy mildew
sp73	Susceptive
sp75	Susceptive
C134	Susceptive
US61	Resistant
US77	Resistant
US78	Resistant
US88	Moderate resistant
US233	Moderate resistant
