# Supplementary material for: Genome-wide identification and expression analysis of nucleotide-binding site leucine-rich repeat genes associated with downy mildew resistance in spinach
Source: PeerJ. 2026 Aug 14;14:e21599. doi: 10.7717/peerj.21599 (PMC13480143; doi:10.7717/peerj.21599)
Supplement: Supplementary file 4 [file peerj-14-21599-s004.pdf]

Table S4 Spinach germplasm material information

Name	Character	Resistance to downy mildew
sp73	sp73（Functional Leaf）	Susceptive
Wild	Wild Leaf	High resistant
Wild	Wild Bolting Petiole	High resistant
US337-9-1	Leaf Division Shallow	High susceptive
US15-3-4-3	Leaf Division Deep	High susceptive
US296-3-5-3	Leaf Creasy	High susceptive
US63-5-9	Leaf Light Green	High susceptive
S14	Leaf Dark Green	High resistant
C318-4-8-3	Leaf Light Red	Moderate resistant
US4-3-5-6	Leaf Dark Red	Moderate resistant
US327-5-3-1	Bolting Early	High susceptive
US185-6-3-1	Bolting Late	High susceptive
